# Development and Clinical Application of a Precise Temperature-Control Device as an Alternate for Conventional Moxibustion Therapy

**DOI:** 10.1155/2012/426829

**Published:** 2012-06-15

**Authors:** Shin Takayama, Shigeru Takashima, Junnosuke Okajima, Masashi Watanabe, Tetsuharu Kamiya, Takashi Seki, Miyako Yamasaki, Nobuo Yaegashi, Tomoyuki Yambe, Shigenao Maruyama

**Affiliations:** ^1^Department of Traditional Asian Medicine, Graduate School of Medicine, Tohoku University, Sendai 980-8575, Japan; ^2^School of Engineering, Tohoku University, Sendai 980-8579, Japan; ^3^Institute of Fluid Science, Tohoku University, Sendai 980-0812, Japan; ^4^Department of Geriatrics and Gerontology, Institute of Development, Aging and Cancer, Tohoku University, Sendai 980-0872, Japan; ^5^Department of Medical Engineering and Cardiology, Institute of Development, Aging and Cancer, Tohoku University, Sendai 980-0872, Japan

## Abstract

Moxibustion therapy has been used in East Asian medicine for more than a thousand years. However, there are some problems associated with this therapy in clinical practice. These problems include lack of control over the treatment temperature, emission of smoke, and uneven temperature distribution over the treatment region. In order to resolve these problems, we developed a precise temperature-control device for use as an alternate for conventional moxibustion therapy. In this paper, we describe the treatment of a single patient with paralytic ileus that was treated with moxibustion. We also describe an evaluation of temperature distribution on the skin surface after moxibustion therapy, the development of a heat-transfer control device (HTCD), an evaluation of the HTCD, and the clinical effects of treatment using the HTCD. The HTCD we developed can heat the skin of the treatment region uniformly, and its effect may be equivalent to conventional moxibustion, without the emission of smoke and smell. This device can be used to treat ileus, abdominal pain, and coldness of abdomen in place of conventional moxibustion in modern hospitals.

## 1. Introduction

 In East Asian medicine, a local thermal therapy known as moxibustion is widely used to treat several conditions, including gastrointestinal disorders, neurodegenerative diseases, cerebrovascular diseases, and cardiovascular diseases [1–3]. In moxibustion therapy, skin is heated by burning moxa. Because the direct burning of moxa on the skin can be dangerous, materials like salt, ginger, or garlic may be used as a buffer between the skin and the moxa [4–7]. There have been several studies reporting the effects of moxibustion therapy [4–7]. However, there are some problems associated with the application of this treatment in clinical practice. Moxibustion treatment temperature is dependent upon the expertise of the treating doctor or practitioner. Thermal damage to tissue begins at temperatures above 44°C [8]. Therefore, patients who have lost temperature sensitivity are at risk for burns during moxibustion therapy. A uniform temperature distribution is important when conducting thermal therapy in order to avoid burning the patient and to heat the treatment region to the desired therapeutic temperature. Additionally, the temperature distribution in the treatment region is nonuniform. Another problem associated with moxibustion therapy is the emission of smoke. In order to resolve these problems, we developed a precise temperature-control device for use as an alternate for conventional moxibustion therapy.

 In this paper, we describe a case of repetitive ileus that was treated by moxibustion. We also describe an evaluation of temperature distribution on the skin surface after conventional moxibustion therapy, the development of a heat-transfer control device (HTCD) for use as an alternate for conventional moxibustion therapy, an evaluation of the HTCD, and the clinical effects of the HTCD.

## 2. Clinical Usage of Moxibustion to Treat Ileus: A Case Report

**Figure 1:**
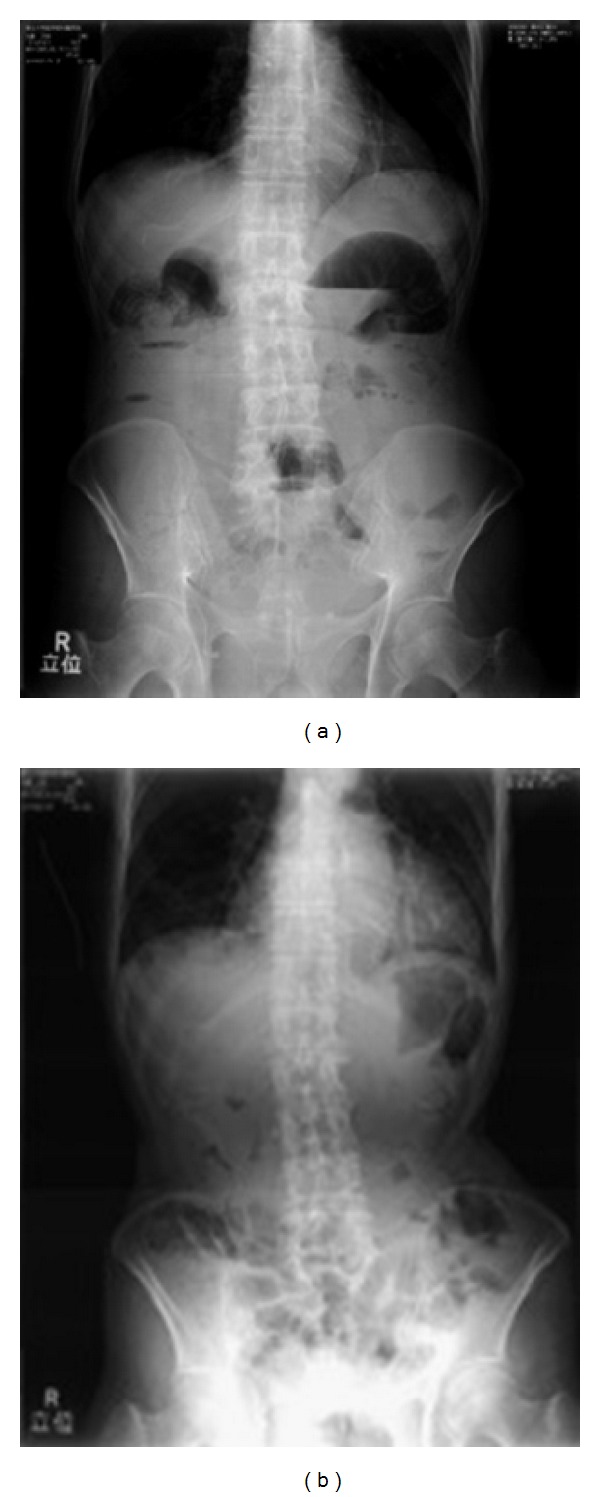
Abdominal X-ray radiographs of a 95-year-old paralytic ileus patient. (a) Radiograph taken at the time of hospital admission, (b) radiograph taken 18 days after moxibustion therapy.

**Figure 2:**
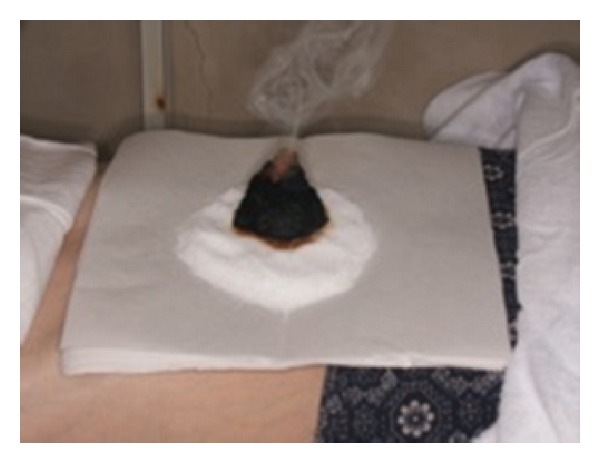
A type of conventional moxibustion therapy. Salt was used as a buffer between the skin and the moxa.

 A 95-year-old man was referred to Tohoku University Hospital from a local clinic for the treatment of nausea and abdominal pain. The patient was admitted to the hospital and diagnosed with paralytic ileus. He underwent an operation for inguinal herniation 15 years before seeking treatment at our hospital and had experienced ileus twice in the preceding year. The patient indicated he had been experiencing symptoms of constipation and abdominal pain for 5 days. After hospitalization, he was prescribed aperients. An abdominal X-ray radiograph indicated gas in the intestine and air-fluid level (Figure 1(a)). There was no indication of tumor or strangulation in either the small or large intestine, and the diagnosis of paralytic ileus was confirmed. Because the patient experienced repeated ileus during treatment with medication, we added moxibustion therapy to his treatment regimen (Figure 2). Following moxibustion therapy around umbilical region for 10 days, the constipation was relieved. The patient no longer experienced nausea and abdominal pain and he was able to resume eating meals. An abdominal radiograph also indicated improved ileus (Figure 1(b)). Moxibustion therapy was repeated daily, and the patient was able to defecate without anticonstipation drugs. After his discharge, the patient continued to receive regular weekly moxibustion therapy in the outpatient clinic.

## 3. Temperature Distribution on the Skin by Conventional Moxibustion (Modified from [9])

**Figure 3:**
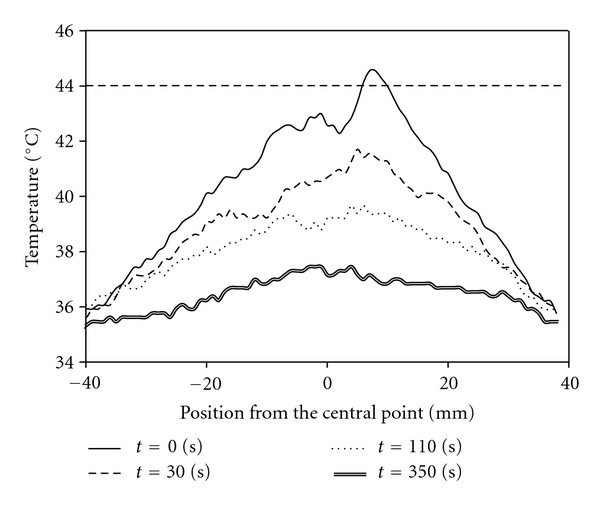
Spatial distribution of abdominal skin temperature from the central point at 0, 30, 110, and 350 s after completion of moxibustion therapy. The way of moxibustion is shown in Figure 2 (modified from [9]).

The temperature distribution on the skin by moxibustion should be assessed to avoid the skin burning. Therefore, we investigated temperature distribution on the skin after conventional moxibustion therapy in human.  Figure 3 shows the variation over time in the temperature distribution on the skin surface measured using thermography (Advanced Thermo TVS-500^®^; Nippon Avionics Co., Ltd., Tokyo, Japan) just after conventional moxibustion therapy (Figure 2). In this experiment, the abdominal area of 34-year-old healthy male volunteer was heated by moxibustion with a salt buffer between the skin and moxa. As shown in Figure 3, the temperature on the central part of the abdominal area reached 44°C, indicating the risk of burns from heating the skin for long periods of time, because the denaturation of protein occurs at temperatures greater than 44°C [8]. In moxibustion therapy, the treatment temperature is adjusted based on the expertise of doctors and feedback from patients. Therefore, patients who have lost heat sensitivity like aged person are at increased risk of burns during moxibustion therapy. It is safer to control temperature precisely when the treatment is applied to human.

## 4. Development of an HTCD (Modified from [9])

**Figure 4:**
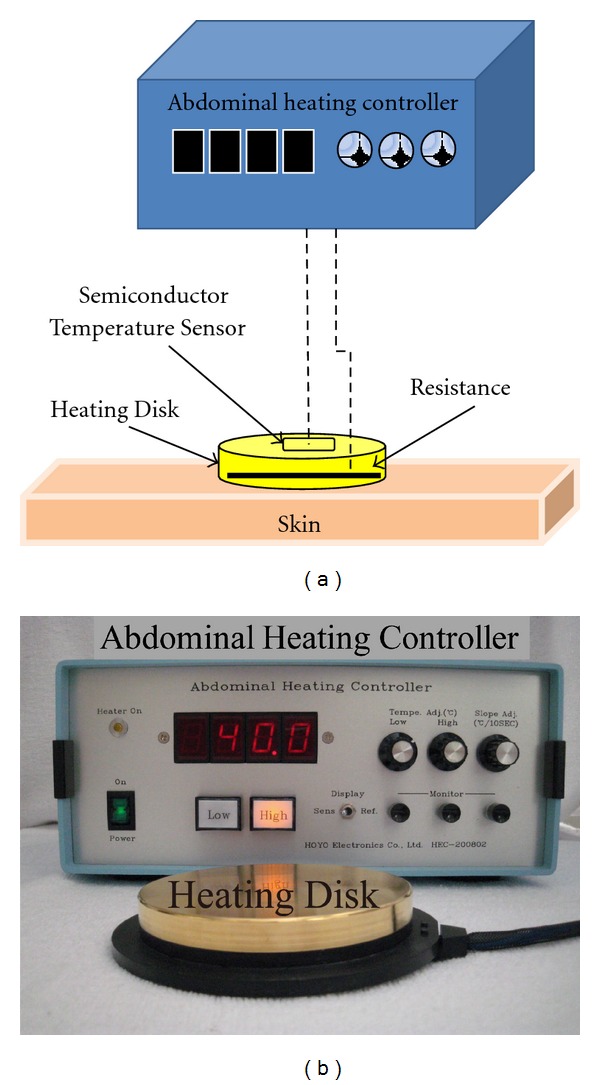
(a) Schematic diagram of an abdominal heating controller, (b) picture of heat-transfer control device.

 According to the data of temperature distribution on the skin and the style of conventional moxibustion, we have developed a precise temperature control devise. A schematic diagram of the HTCD is provided in Figure 4. This device consists of heating disk and a control apparatus. The copper heating disk is 100 mm in diameter. Copper was used to form the heating disk because the high thermal conductivity of this material permitted uniform heating of the disk. A thermistor was implanted into the heating disk in order to monitor its temperature within 0.1°C. This device reduces the risk of burns because the temperature of the heating disk can be controlled to avoid exceeding the target temperature. This device is convenient for medical doctors because it does not emit smoke, control of device temperature is easy, and there is no risk of burns to patients. Therefore, this devise as a modern “moxibustion” therapy can be used in hospitals. Furthermore, we have demonstrated the therapeutic effects of this device on many patients.

## 5. Temperature Distribution on the Skin by HTCD

### 5.1. Methods (Modified from [9])

**Figure 5:**
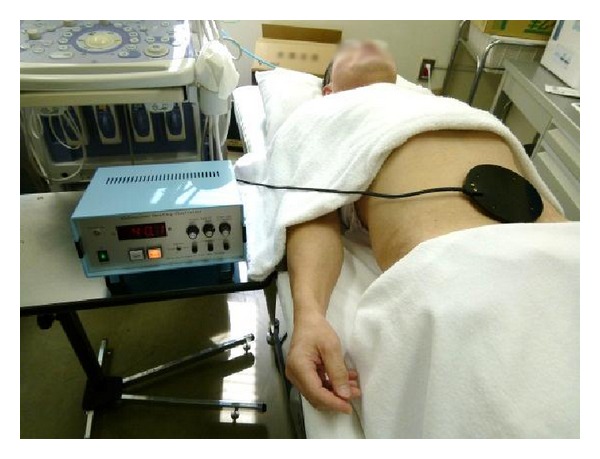
Abdominal thermal therapy on the abdomen by using a heat-transfer control device.

To confirm the performance of the abdominal heating controller, we evaluated the controllability of the temperature, heating rate, and uniformity of the temperature distribution on the skin surface. A 24-year-old healthy male volunteer lay down on his back on a bed in the experiment room. The temperature of the experiment room was maintained at 25°C. The HTCD was placed directly on the skin of the subject's abdominal area (Figure 5). The temperature of heating disk was set to 42°C and the abdominal area was heated for about 12 minutes. Skin surface temperature was recorded by temperature sensor in the disk. The experiment was repeated 4 times with the interval of 2 hours and in each experiment, the incremental rate of temperature increase was altered. After heating for 12 minutes, the heating disk was removed from the subject's abdomen and the temperature distribution of the skin surface was measured using thermography.

### 5.2. Results

**Figure 6:**
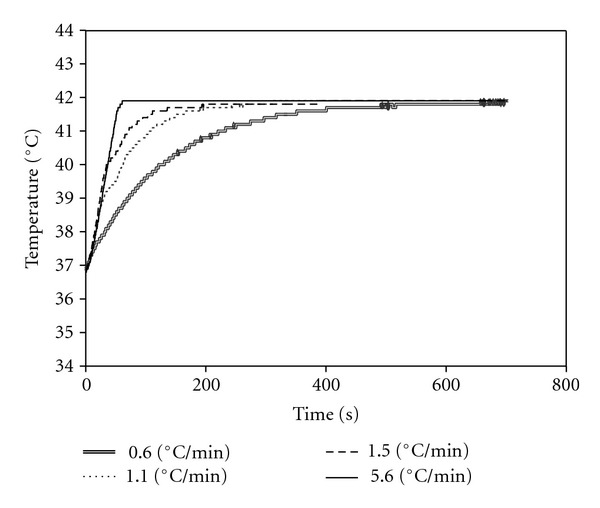
Skin surface temperature of a single subject during 12 minutes of stimulation by a heat-transfer control device set to 42°C. The device was set to increase from body temperature to the target temperature at the following rates: 0.6°C/min, 1.1°C/min, 1.5°C/min, and 5.6°C/min (modified from [9]).

**Figure 7:**
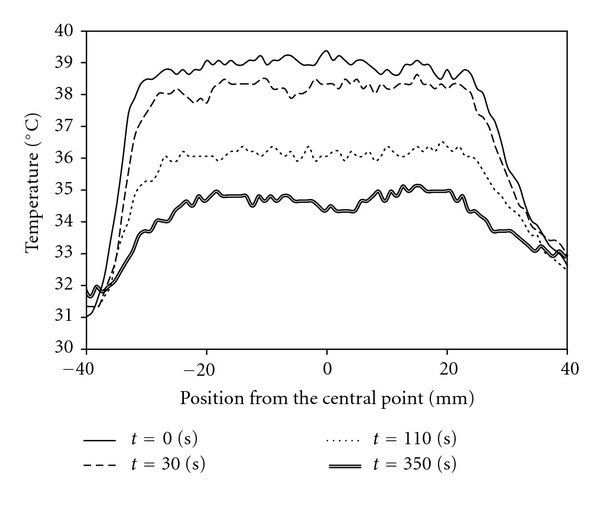
Spatial distribution of abdominal skin temperature after stimulation with a heat-transfer control device. The target temperature was set at 42°C. Measurements were taken 0, 30, 110, and 350 seconds after device removal. (Modified from [9]).

Figure 6 shows the result in the variation of the incremental rate of temperature on the skin surface of the subject's abdominal area. In all 4 repetitions of the experiment, the temperature of the heating disk increased to the target temperature, but did not exceed it. This confirms that there is no risk of skin burns to patients from high temperatures. Moreover, it was confirmed that the temperature of the disk is controllable. The temperature distribution on the skin at different time points after the removal of a heating disk set to 42°C is shown in Figure 7. Skin temperature decreased almost uniformly. This confirms that the temperature distribution of the heating disk and that of the subject's abdominal area were almost uniform during the procedure. Therefore, the HTCD is able to uniformly heat the abdominal area of patients to a precise target temperature.

## 6. Clinical Application [10, 11]

### 6.1. Methods

 To confirm the effects and safety of HTCD, the thermal therapy was conducted on 26 healthy volunteers (male; 24 and female; 2) with a mean age of 30.8 ± 6.8 years (range, 21–44 years). The HTCD was placed on the skin of the paraumbilical region for 20 minutes. We measured blood flow through the superior mesenteric artery (SMA) continuously from rest to 40 minutes after removal of the HTCD. (Figure 5). There are several acupoints located at the paraumbilical region. In particular, the acupoint of CV8 and ST25 is considered to influence the stomach, spleen, and intestines in traditional East Asian medicine [12]. This size of the heating disk can cover these important acupoints for the treatment of digestive diseases. The SMA supplies blood to the whole small intestine, except the superior part of the duodenum, as well as the cecum, the ascending colon, and most of the transverse colon. Therefore, we used SMA blood flow to investigate the changes in intestinal blood flow resulting from thermal stimulation. We measured circulation in the SMA using an ultrasound system (Prosound *α*10^®^; Aloka Co., Ltd, Tokyo, Japan). Pulsed Doppler signals were used to acquire SMA measurements within 2 to 3 cm of the origin of the artery [13, 14]. To ensure accurate measurement, we employed a Doppler angle of 60° or less [15, 16]. Blood flow volume was recorded 3 times during 3 different cardiac cycles and averaged for each subject in an effort to minimize errors [13].

 Subjects rested in the supine position in a quiet, air-conditioned room (temperature, 25-26°C) during the entire experiment. After positioning the ultrasound system, subjects rested for 10 minutes. The HTCD was set to 40°C and placed on the skin of the paraumbilical region (Figure 5). The HTCD was left in place for 20 minutes. After 5 minutes, if tolerated by subjects, the temperature was increased to 41°C. The target temperature of the disk was set at 40-41°C for safety to avoid a skin burn. The device was removed after 20 min of thermal stimulation.

 Statistical analysis was performed with SPSS software (version 16.0, SPSS Japan Inc., Tokyo, Japan). Repeated measures analysis of variance with a Tukey post hoc test was used for statistical comparison with baseline. The % change of blood flow volume in the SMA at each time was calculated in relation to the baseline value. Results are presented as the means and SD. *P* < 0.05 was used to indicate significance in all statistical tests.

### 6.2. Results

**Figure 8:**
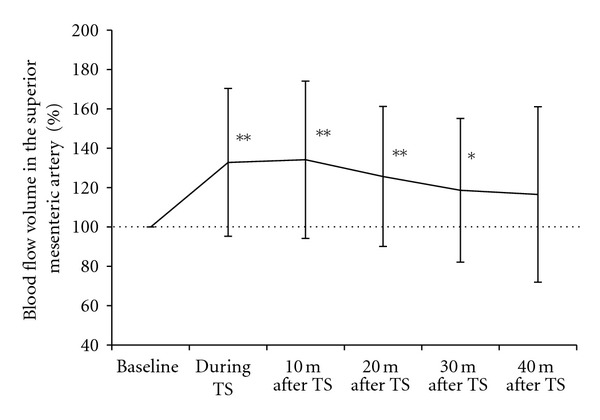
The % change of blood flow volume in the superior mesenteric artery during and after thermal stimulation of the abdomen. Data are presented as means and SD. *P < 0.05, **P < 0.01 versus baseline. TS, thermal stimulation.

Figure 8 shows the result in the % change of blood flow volume in the SMA. The blood flow volume in the SMA was significantly higher during thermal stimulation (*P* < 0.01), 10 and 20 minutes after the end of thermal stimulation (*P* < 0.01), and 30 minutes after the end of thermal stimulation (*P* < 0.05), as compared to the volume before placement of the HTCD (Figure 8). There were no complications such as local burns, pain, discomfort or other problems that required treatment.

## 7. Discussion

 In this report, we described a case of ileus that was successfully treated with moxibustion, reported the distribution of skin temperature following conventional moxibustion, described the development of a precise temperature-control device, and reported the results of an experiment demonstrating the efficacy of the devise.

 Traditional East Asian medicine has a long history and the administration of herbal medicine, acupuncture, and moxibustion therapies depends on the experience of the treating doctors or practitioners. The problems associated with moxibustion therapy, such as difficulty of temperature control, smoke, and smell make it difficult to use in modern hospitals. These problems pose real risks to patients. In general clinical practice, there is a possibility of skin burning of a patient with low temperature sensitivity during moxibustion. Another patient suffered an attack of bronchial asthma during moxibustion therapy that was induced by the smoke.

 The effects of moxibustion result from the heat, smell, and smoke generated by the procedure. The temperature-control device that we described in this study simulates the heat of moxibustion, but does not produce either the smoke or smell associated with this treatment. As this device can be used to control skin surface temperature to within 0.1°C, it can be used in modern hospitals as a safer and more generally useful device than conventional moxibustion.

 Mesenteric ischemia results from decreased blood flow to the bowels and causes several symptoms such as pain, nausea, and vomiting. Nonocclusive mesenteric ischemia is an acute mesenteric circulatory disorder, which is induced by vasospasm [17]. Chronic mesenteric ischemia is usually caused by atherosclerosis [18]. In these conditions, the pathophysiology is same as the mesenteric ischemia. The treatment of mesenteric ischemia involves reperfusion through drug therapy or vessel reconstruction. Sometimes vasodilative drugs are selected as a conservative treatment modality [17–19]. In the current study, we demonstrated that SMA blood flow volume increases with local thermal therapy. The treatment of local thermal therapy with HTCD might be useful for increasing blood flow volume in the cases of mesenteric ischemia. During thermal stimulation, intestinal peristalsis accelerated along with the increase in blood flow volume (data not shown). Thus, thermal stimulation not only increases the blood flow volume, but also improves intestinal motility. Abdominal thermal therapy might be useful for patients with low SMA blood flow, paralytic ileus, or chronic constipation. Future study of the effect of thermal stimulation on patients with such disorders is warranted.

 HTCDs have been used to treat the abdominal disorders, pain, and coldness in about a total of 600 patients at Tohoku University Hospital. Over the past 3 years, there have been no reported side effects associated with the use of this device. We conduct clinical trials to investigate its clinical effects in the patients of old cerebral infarction with chronic constipation.

## 8. Conclusion

 The HTCD described in this paper was developed based on conventional moxibustion therapy. The temperature of this device can be controlled precisely, and it is safer than conventional moxibustion. Our studies suggest the usefulness of this device for the treatment of adnominal disorders, constipation, pain syndromes, and coldness in place of moxibustion in hospital settings.
